# Short-term and long-term results after intravitreal bevacizumab therapy for macular oedema in branch retinal vein occlusion

**DOI:** 10.22336/rjo.2022.6

**Published:** 2022

**Authors:** Ajla Pidro, Aida Pidro, Amra Nadarevic-Vodencarevic, Meliha Halilbasic, Orhan Lepara, Faruk Nisic

**Affiliations:** * Policlinic Anda, Zagreb, Croatia; ** Department of Ophthalmology, “Prim. Dr. Abdulah Nakas” General Hospital, Sarajevo, Bosnia and Herzegovina; *** Department of Ophthalmology, Tuzla University Clinical Center, Tuzla, Bosnia and Herzegovina; **** Institute of Physiology and Biochemistry, Faculty of Medicine, University of Sarajevo, Sarajevo, Bosnia and Herzegovina; ***** Clinic for Eye Disease, Sarajevo Clinical Centre University, Sarajevo, Bosnia and Herzegovina

**Keywords:** retinal vein occlusion, macular oedema, bevacizumab

## Abstract

**Objectives:** The aim of this study was to determine the possible correlation between the short- and long-term effects of intravitreal bevacizumab on macular oedema after branch retinal vein occlusion (BRVO).

**Material and methods:** This prospective clinical study included fifteen eyes of patients with macular oedema after BRVO. Corrected distance visual acuity (CDVA), recorded in LogMAR units, central foveal thickness (CFT) and maximum foveal thickness (MFT) were evaluated at one month after first application and at least every 2 months for one year. PRN treatment protocol was used for all patients. Statistical calculation was performed with SPSS for Windows and Microsoft Excel.

**Results:** Mean CFT decreased significantly (p<0,0001) from baseline 471,2 ± 151,7 μm to 285,9 ± 79,82 μm at 12 months. CDVA improved significantly (p<0,0001) from baseline 0,58 ± 0,34 to 0,1 ± 0,25 at the end of follow up period. Change from baseline in the CDVA after one month was significantly positively correlated with the change in CDVA after 12 months (r=0,76, p=0,001). Change in CFT after one month had a strong positive correlation (r=0,78, p=0,001) with change after 12 months. There was no statistically significant correlation between the number of injections and the changes in CDVA, CFT, MFT after a single injection.

**Conclusions:** Single injection effects of bevacizumab may indicate long-term results on macular oedema after BRVO, but further and larger studies are necessary.

**Abbreviations:** BRVO = Branch retinal vein occlusion, RVO = Retinal vein occlusion, CFT = Central foveal thickness, MFT = Maximum foveal thickness, VEGF = Vascular endothelial growth factor, MO = Macular oedema, CDVA = Corrected distance visual acuity, PRN = Pro-re-Nata, SD-OCT = Special-domain optical coherence tomography, FT = Foveal thickness, LogMAR = Logarithm of the Minimum Angle of Resolution, WHO = World Health Organization, RPE = Retinal pigment epithelium

## Introduction

Branch retinal vein occlusion (BRVO) is an acute ophthalmological condition with a high exposure of intravitreal endothelial growth factor (VEGF), which correlates with macular oedema (MO) degree and the visual acuity decrease [**1**]. The treatment choice is anti-VEGF therapy that reduces intraretinal fluid levels [**2**,**3**]. This study described a 12-month follow-up of patients who received intravitreal bevacizumab due to MO secondary to BRVO. The aim was to determine a correlation between a short-term and a long-term effect of intravitreal bevacizumab on MO and to determine if short-term results can be used as a predictor for a total number of injections. 

## Material and Methods

This is a prospective clinical study performed over a period of 3 years, from august 2014 to august 2017. It included 15 eyes in total, 8 of males and 7 of females. The follow-up period was 12 months and included evaluation one-month and at least every two months for one year. 

Record data and outcome measures included CDVA, CFT and maximum foveal thickness (MFT) one month, six months and 12 months post first injection, which were later compared to baseline. Total number of required injections was also recorded. 

Treatment included intravitreal 1.25 mg bevacizumab application following Pro-re-Nata (PRN) protocol, treating the patient with injections in a case of an active disease (FT 300 µm). 

Each exam included CDVA measurement recorded in LogMAR units for statistical analysis, slit-lamp anterior and posterior segment examination using pan fundoscopic lens and CFT and MFT measurement on special-domain optical coherence tomography (SD-OCT). 

Inclusion criteria were MO involving the fovea secondary to BRVO with baseline FT ≥ 300 µm, patients who were just diagnosed and not previously treated and patients who used anti-VEGF injections other than bevacizumab. Exclusion criteria were patients who did not attend regular follow-ups or did not give an informed consent, patients with previous history of intravitreal or corticosteroid injections or other MO causes, presence of other retinal disease or previous vitreoretinal surgery.

Informed consent was obtained from all patients included in the study, conforming to local laws and in compliance with the principles of the Declaration of Helsinki and WHO guidelines. Statistical calculation was performed with SPSS for Windows (19.0, SPSS Inc, Chicago, Illinois, SAD) and Microsoft Excel (11.0, Microsoft Corporation, Redmond, WA, SAD). Comparison between follow-ups was performed with the Wilcoxon signed rank test, correlations with Pearson correlation. Value of p<0.01 was considered statistically significant.

## Results

Out of all the patients treated, 15 patients (15 eyes) presented a complete follow up and were included in the study. Baseline values are presented in **Table 1**. Five patients underwent laser photocoagulation during the study, due to large areas of non-perfusion and (one developed neovascular vessel on mid-periphery). No complications (IOP spikes, endophthalmitis or retinal holes) that can be associated with the treatment were reported. 

**Table 1:** Baseline characteristics (n=15)

Age (years)	59.9 ± 7.8
Gender (male/ female)	8/ 7
Symptom duration (months)	1.6 ± 1.09
CDVA (logMAR)	0.58 ± 0.34
Baseline MFT (μm)	588 ± 144
Baseline CFT (μm)	471 ± 152
Systemic hypertension presence	11 (73.3%)
Diabetes mellitus presence	3 (20%)
Legend: n = number, CDVA = Corrected distance visual acuity, MFT = Mean foveal thickness, CFT = central foveal thickness	

The mean number of injections in this study was 3.28 ± 1.92, minimum being one injection (in one patient, or 6,6%), and maximum being 7 injections (in 2 patients, 13,3%).

**Fig. 1:**
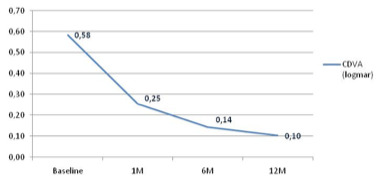
Mean CDVA at each follow-up evaluation (n=15) CDVA = corrected distance visual acuity, M = month, n = number

CDVA improved significantly after the application of anti-VGF therapy. The biggest change and improvement occurred after the first injection, and continued through the follow-up period, improving to 0.10 ± 0,25 at the end of the study. Increase in SD indicated that the distribution of the results was more scattered than at baseline, and that the therapy might have had a different effect on some patients. The mean CDVA through follow-up is presented in **Fig. 1** (p < 0.005).

**Fig. 2:**
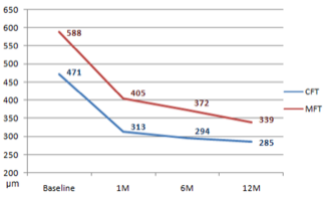
CFT and MFT at each follow-up evaluation (n=15) (p<0.005) CFT = central foveal thickness, MFT = maximum foveal thickness

Both CFT and MFT decreased significantly (p < 0.0001) 1 month after the first injection. Significant reductions continued at 1, 6 and 12 months (p < 0.005) (**Fig. 2**). 

The biggest drop was after one month, which was in an accordance with the biggest improvement in visual acuity at this point. By the end of the follow up period CTF was 285 ± 80 µm, and MFT 340 ± 84 s µm, which was a statistically significant decrease from the baseline (p < 0.0001).

Change from CDVA baseline after one month (ΔVA-1M) was significantly positively correlated with the change in CDVA after 12 months (ΔVA-12 M; r =0,76, p=0,001). Change in CFT after one month had a strong positive correlation (r=0,78, p=0,001) with change after 12 months. There was no statistically significant correlation between the number of injections and changes in CDVA, CFT, MFT after a single injection.

## Discussion

This article evaluated the results to treatment of MO secondary to RVO with bevacizumab, following PRN protocol. PRN protocol and bevacizumab were used in this study because monthly visits, the high number of injections and more expensive medicine represent a burden for many patients. This is a reality in all less developed countries, “adjusting” the protocols and “extending” the period between the injections and providing the best health care, usually using as few resources as possible, compared to strict protocols in most studies. 

The average age in the study was 59.9 ± 7.8 years. Age was strongly correlated to RVO, where older age was correlated to be the risk factor for the RVO incident, and younger age, together with good CDVA, was a favorable prognostic sign [**4**,**5**]. 

Two risk factors were documented in this study: systemic hypertension and diabetes mellitus presence. There were significantly more patients with systemic hypertension (73.3%) than with diabetes (20%), suggesting that hypertension is the leading cause of RVO and MO [**4**].

The mean number of received injections was 3.28 ± 1.9 in the period of 12 months, which was lower compared to some other studies. Jumper et al. reported the average number of total injections, which was 7.1 during the first year of therapy [**3**]. This study had different inclusion criteria and included patients who had already received three anti-VEGF intravitreal injections and RVO associated MO, but still our protocol allowed a lower number of injections with good visual results. Wecker et al. reported 6 average total injections in the first year of treatment, using a treatment protocol as-needed [**6**]. On the other hand, Wang et al. and Kiss et al. reported a mean injection number of bevacizumab 2.22 and 2.5 respectively during the first-year studies, which is comparable to our results. This led to the conclusion that the number of injections is lower following PRN protocols, than three-loading and PRN groups as in most other [**7**,**8**].

Goals and endpoint of this study of MO treatment with anti-VEGF was both, decreasing MO and subretinal fluid and increasing CDVA. Our study showed an improvement in both parameters from the baseline values that sustained during a follow-up period. As expected, there has been an improvement in CDVA after anti-VEGF therapy. The first injection showed the biggest improvement in CDVA, which was consistent with other real-life studies [**2**]. CDVA improvement after a follow-up period was 0.1 log-Mar ± 0.25, which was higher compared to other studies, this study having a higher number of patients. Lower visual acuity in those studies can also be due to lower baseline visual acuity [**2**,**9**]. Lower average number of injections can also cause inferior increase in CDVA, due to the progression of ischemia, RPE atrophic changes and progressive cell apoptosis [**2**,**5**]. 

The combination therapy with laser photocoagulation or dexamethasone implant or even a switch to another anti-VEGF in non-responder can also affect the results in a form of less frequent MO relapses and therefore more stable retina [**10**]. Our study included 5 patients with additional laser photocoagulation due to large non-perfusion areas.

CFT and MFT reflected an improvement as CDVA. There was a significant decrease in both CFT (from 471 ± 152 μm to 285 ±80 μm) and MFT (from 588 ± 144 to 340 ± 84 μm) after the first injection, and continued at one, six and twelve months after the treatment and additional injections. The biggest improvement was after the first injection. These results correlate with other studies and they also remain stable following PRN protocol [**2**,**11**,**12**]. Roy et al. reported a better visual outcome in eyes that had more than 25% of CFT reduction, which correlated to our results [**13**]. They also reported that the response to the first injection could have a predictive value for the outcome, which again correlated to our study [**13**].

There is a strong significant positive correlation in the change of both CDVA and CFT baseline after one and after 12 months. Positive correlation can be used to predict the long-term effect based on a short-term result. Minami et al. reported a positive significant correlation in CDVA, unlike FT, which was not significantly correlated, meaning that it could not be used as a long-term predictor [**12**]. The reason for this discrepancy is because Minami et al. measured CDVA and CFT one day after the injection [**12**].

The number of injections did not show a statistically significant correlation to change in CDVA, CFT and MFT after a single injection. The average symptom duration was 1.6 ± 1.09 months prior the first examination and the first injection. Good results could be associated with an early start of therapy, since older occlusions might alter retinal structures and cause a poor response to therapy [**10**,**14**]. Minami et al. also reported a better visual outcome and long-term effects when started sooner [**12**]. Rezar et al. reported that functional recovery, as well as prevention of irreversible damage were better if treatment was started less than three months before the treatment initiation [**11**]. 

The strength of this study was that it was a prospective real-life study, where the examination, diagnostics, injections, and follow-up periods were all organized and funded by patients themselves. Many patients were excluded from the study due to poor compliance. This study also reported the correlation between short and long-term results of bevacizumab therapy. Limitations to this study were the small sample number and a short follow-up period, but it could be used as a pilot study to a large prospective study, which would include a higher number of patients and a longer period of follow-up (two, three and five years). Also, a new cohort study could be made comparing different protocol types and its short and long-term results, or introducing a control group to make a correlation with spontaneous regression. 

## Conclusions

Bevacizumab is a safe and effective treatment for MO caused by RVO. This study proved that the reduction of MO and improvement of CDVA could be expected in patients with an early treatment and a regular follow-up. Further research and a longer follow-up period can be used to strongly confirm these results. 

**Conflict of Interest statement**

Authors state no conflict of interest.

**Informed Consent and Human and Animal Rights statement**

Informed consent has been obtained from all individuals included in this study.

**Authorization for the use of human subjects**

Ethical approval: The research related to human use complies with all the relevant national regulations, institutional policies, is in accordance with the tenets of the Helsinki Declaration, and has been approved by the review board of “Prim. Dr. Abdulah Nakas” General Hospital, Sarajevo, Bosnia and Herzegovina.

**Acknowledgements**

None to declare.

**Sources of Funding**

All the authors involved in this study had no conflict of interest and did not receive funding from any source. No special grant was allotted for this study.

**Disclosures**

None to declare.
